# Unexpected hypotension in catecholamine reversal: a case report

**DOI:** 10.1186/s13256-017-1442-9

**Published:** 2017-10-06

**Authors:** Yohei Okada, Wataru Ishi, Hiromichi Narumiya, Ryoji Liduka

**Affiliations:** Department of Emergency and Critical Care Medicine, Japanese Red Cross Society Kyoto Daini Red Cross Hospital, 355-5 Haruobicho Kamigyoku, Kyoto, 602-8026 Japan

**Keywords:** Adrenaline reversal, Alpha-adrenergic blockade, Vasopressin, Noradrenaline, Side effect, Risperidone, Case report

## Abstract

**Background:**

Catecholamine agents are commonly used to support circulation; however, they may cause unexpected hypotension in a special situation. Here we describe the first unexpected case of hypotension in response to catecholamine agents.

**Case presentation:**

A 29-year-old Japanese man with schizophrenia was transferred to our emergency department. He was in shock and in coma. After fluid resuscitation, we induced catecholamine agents; however, his blood pressure decreased to 59/40 mmHg in response to catecholamine infusion. On the other hand, after we started vasopressin, his blood pressure markedly improved, and he finally became stable. On day 2, he admitted to ingesting a large amount of risperidone, and we diagnosed risperidone overdose. We believe that this unexpected hypotension in response to catecholamine infusion was caused by an α-adrenergic blockade effect of risperidone. Animal experiments proved that the simultaneous administration of adrenaline with an α-adrenergic blockade provoked a fall in blood pressure; this phenomenon is called “adrenaline reversal.” In our case, catecholamine infusion under the α-adrenergic blockade effect of risperidone might have caused a fall in blood pressure in the same mechanism; we call this phenomenon “catecholamine reversal.” In such a situation, because the mechanism of vasopressin is different from that of catecholamine, we recommend vasopressin for maintaining the blood pressure.

**Conclusions:**

We described the first clinical case of “catecholamine reversal” and highlighted that if unexpected hypotension occurs in response to catecholamine infusion, we should suspect the use of α-adrenergic antagonists. In such situations, we should consider the administration of vasopressin instead.

## Background

**Fig. 1:**
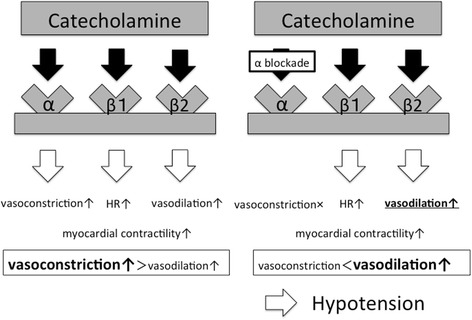
Alpha-adrenergic and β-adrenergic effects of vasoactive catecholamines. Alpha-adrenergic receptors promote peripheral vasoconstriction, β_1_-adrenergic receptors increase chronotropic and inotropic effects, and β_2_-adrenergic receptors increase vasodilation. If α-adrenergic receptor antagonists are simultaneously administered with catecholamine agents, α-adrenergic effects are masked, and β-adrenergic effects are predominantly enhanced. Consequently, vasodilation occurs and blood pressure decreases. *HR* heart rate

Catecholamine is commonly used to support circulation, and each catecholamine agent has different effects on different receptors. Catecholamine action on α-adrenergic receptors promotes peripheral vasoconstriction, on β_1_-adrenergic receptors it increases chronotropic and inotropic effects, and on β_2_-adrenergic receptors it increases vasodilation (Fig. 1) [1]. In distributive shocks such as septic shock, we generally use catecholamine agents, including noradrenaline and dopamine, to promote vasoconstriction because they mainly affect the α-adrenergic receptor [1]. However, in a special situation, these catecholamine agents may cause unexpected hypotension. Here we describe a case of unexpected hypotension in response to the use of catecholamine agents.

## Case presentation

**Fig. 2:**
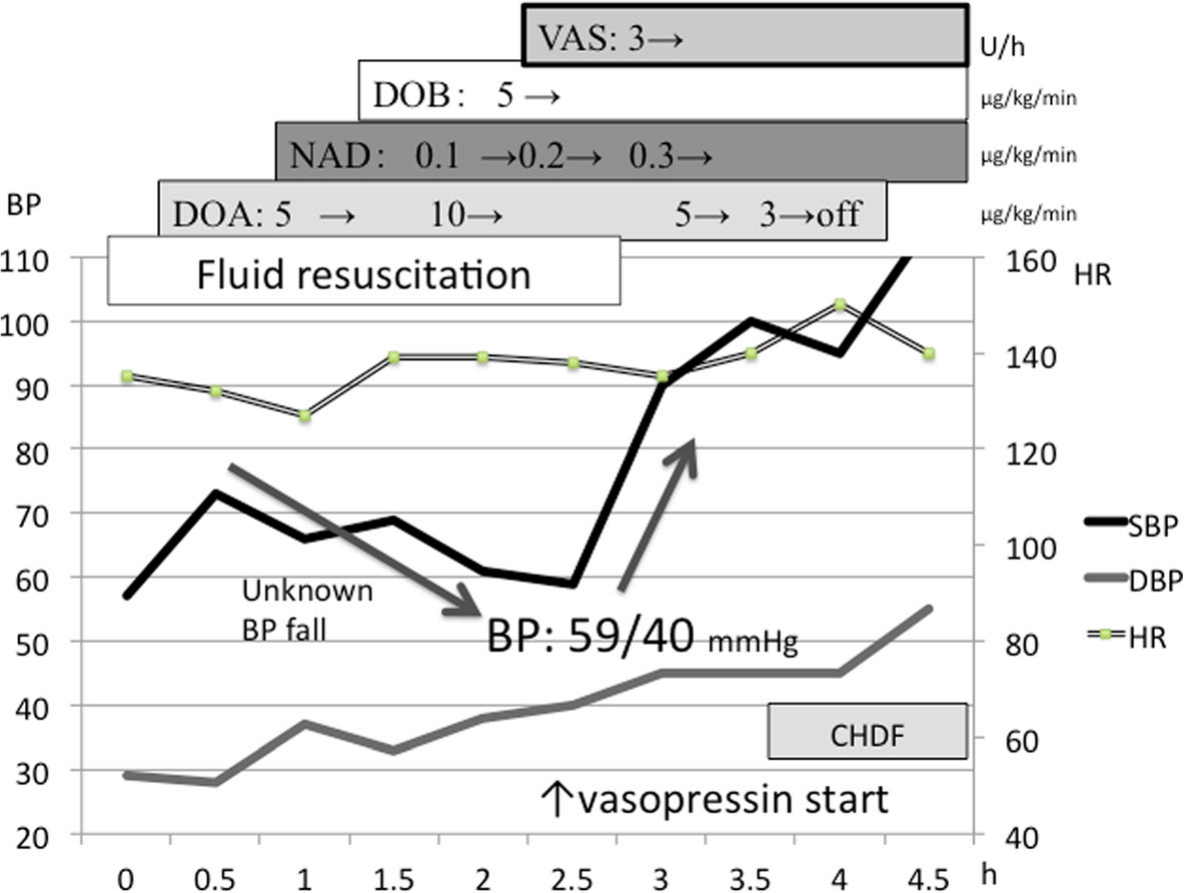
Clinical course after admission. Blood pressure gradually decreased in response to an increase in catecholamine administration. After initiating vasopressin, hypotension markedly improved. *BP* blood pressure, *CHDF* continuous hemodiafiltration, *DBP* diastolic blood pressure, *DOA* dopamine, *DOB* dobutamine, *HR* heart rate, *NAD* noradrenaline, *SBP* systemic blood pressure, *VAS* vasopressin

A 29-year-old Japanese man with schizophrenia was transferred to our emergency department. On arrival, he presented with shock and coma. His blood pressure (BP) was 57/29 mmHg, heart rate (HR) was 135 beats per minute (bpm), respiratory rate was 40/minute, and his body temperature was 35.6 °C. His Glasgow Coma Scale score was (E1V1M1) 3. We immediately performed intubation because of his shock and coma. Fluid resuscitation of 3000 ml crystalloid temporarily increased his BP to 73/28 mmHg, but his shock still persisted. Before a central venous line was inserted, we tentatively initiated dopamine infusion at 5 μg/kg per minute, which was increased to 10 μg/kg per minute; however, his hypotension gradually worsened to 66/37 mmHg (Fig. 2). Sixty minutes after arrival, we inserted the central venous line and initiated noradrenaline infusion at 0.1 μg/kg per minute, which was subsequently increased to 0.3 μg/kg per minute. Moreover, 90 minutes after arrival, we initiated dobutamine at 5 μg/kg per minute. However, his BP unexpectedly decreased to 59/40 mmHg. Head computed tomography, enhanced chest-abdominal computed tomography, point of care sonography, and laboratory data (Table 1) did not reveal the cause of coma and hypotension. His systemic vascular resistance index (SVRI) was very low (432 dynes/second/cm/m^2^; normal range, 1970 to 2400 dynes/second/cm/m^2^; Vigileo FloTrac™, Edwards, USA). Thus, we suspected unknown distributive shock refractory to a large amount of catecholamine infusion. Therefore, in addition to catecholamine infusion, we initiated vasopressin at 3 U/hour 150 minutes after arrival. Subsequently, his BP markedly improved to 135/45 mmHg. Three hours after arrival, we transferred him to our intensive care unit. We performed continuous hemodiafiltration because of the presence of metabolic acidosis (Table 2). We gradually decreased the amount of catecholamine infused. Approximately 12 hours after arrival, his SVRI improved to 2271 dynes/second/cm/m^2^ and his hemodynamic state became stable: BP, 140/80 mmHg; HR, 95 bpm. Subsequently, we completely terminated catecholamine infusion. Moreover, we terminated continuous hemodiafiltration because his metabolic acidosis was improved: pH, 7.386; partial pressure of oxygen in arterial blood (PaO_2_), 73.8 mmHg; partial pressure of carbon dioxide in arterial blood (PaCO_2_), 39.3 mmHg; bicarbonate (HCO_3_^-^), − 1.2 mmol/l; lactate (Lac), 1.5 mmol/l; and fraction of inspired oxygen (FiO_2_), 0.3. On day 2, we terminated vasopressin infusion. After extubation, his condition was stable: BP, 123/75 mmHg; HR, 100 bpm; and Glasgow Coma Scale score (E4V5M6), 15. He was transferred to our general ward. He admitted to ingesting approximately 150 mg risperidone to attempt suicide. The risperidone concentration in his blood sample on admission was very high (398 ng/ml at admission, recommended therapeutic range, 20 to 60 ng/ml [2]), which decreased to 3.60 ng/ml on day 2. Consequently, we diagnosed risperidone overdose. Subsequently, his condition was stable without any event, and he was transferred to a psychiatric ward for psychiatric care on day 5.

**Table 1:** Laboratory data on admission

Complete blood count		Coagulation	
WBC	5600/μl	PT-INR	1.09
RBC	458 × 10^4^/μl	APTT	23.9
Hb	13.1 g/dl	Fib	218 mg/d
Ht	40.3%		
Plt	17.9 × 10^4^/μl	Arterial blood gas	
Chemistry		pH	7.364
AST	21 U/L	pCO_2_	36.8 mmHg
ALT	16 U/L	pO_2_	483 mmHg
CPK	136 U/L	HCO_3_^-^	20.4 mmol/l
Cr	1.21 mg/d	Base excess	−3.9 mmol/l
BUN	15.2 mg/d	Lac	4.9 mmol/l
Na	141 meq/l		(FiO_2_ 1.0)
K	2.7 meq/l		
Cl	103 meq/l		
CRP	0.08 mg/d		
PCT	0.03 ng/d		

*ALT* alanine aminotransferase, *APTT* activated partial thromboplastin time, *AST* aspartate aminotransferase, *BUN* blood urea nitrogen, *Cl* chlorine, *CPK* creatine phosphokinase, *Cr* creatinine, *CRP* C-reactive protein, *Fib* fibrinogen, *FiO*_*2*_ fraction of inspired oxygen, *Hb* hemoglobin, *HCO*_*3*_^*-*^ bicarbonate, *Ht* hematocrit, *K* potassium, *Lac* lactate, *Na* sodium, *pCO*_*2*_ partial pressure of carbon dioxide, *PCT* procalcitonin, *pH* potential of hydrogen, *Plt* platelets, *pO*_*2*_ partial pressure of oxygen, *PT-INR* prothrombin time-international normalized ratio, *RBC* red blood cells, *WBC* white blood cells

**Table 2:** Arterial blood gas analysis in intensive care unit

PaCO_2_	34.8 mmHg
PaO_2_	214 mmHg
HCO_3_^-^	14.6 mmol/l
Base excess	−11.4 mmol/l
Na^+^	141 mmol/l
K^+^	2.8 mmol/l
Cl^-^	107 mmol/l
Lac pH	12.6 mmol/l 7.246
(FiO_2_ 0.6)	

*Cl* ^*-*^ chloride ion, *FiO*_*2*_ fraction of inspired oxygen, *HCO*_*3*_^*-*^ bicarbonate, *K* ^*+*^ potassium ion, *Lac* lactate, *Na* ^*+*^ sodium ion, *PaCO*_*2*_ partial pressure of carbon dioxide in arterial blood, *PaO*_*2*_ partial pressure of oxygen in arterial blood, *pH* potential of hydrogen

## Discussion

We experienced unexpected hypotension in response to catecholamine infusion, and we believe that this unexpected hypotension was caused by a pharmacological phenomenon: the catecholamine effect under the α-adrenergic blockade effect of risperidone. In animal experiments, if adrenaline is simultaneously administered with α-adrenergic receptor blockers such as phentolamine, the α-adrenergic effects are masked and the β_2_-adrenergic effects are predominantly enhanced (Fig. 1) [3]. Consequently, vasodilation occurs and the BP decreases. This unique phenomenon is called “adrenaline reversal” [3]. Adrenaline reversal has also been reported in clinical situations; paradoxical hypotension due to adrenaline infusion has been reported in a case of massive quetiapine overdose because quetiapine has an α-adrenergic blockade effect [4]. This report suggested that adrenaline reversal occurs even in cases of massive antipsychotic overdose. This report recommended selecting noradrenaline for hypotension under an α-adrenergic blockade effect, such as an overdose of quetiapine; however, we disagree with this. This is because other animal experiments proved that noradrenaline could also cause the same phenomenon as “noradrenaline reversal” [5], although noradrenaline has stronger α-adrenergic effects than β-adrenergic effects. Therefore, we suggest that we should avoid noradrenaline in such a situation.

Dopamine and dobutamine also have both α-adrenergic and β-adrenergic effects [6]; we think that there is a possibility that dopamine and dobutamine also may cause catecholamine reversal. Thus, catecholamine agents other than adrenaline can potentially provoke “catecholamine reversal” in patients who have used α-adrenergic antagonists. In our case, because risperidone has an α-adrenergic blockade effect, a large amount of catecholamine infused under the effect of an α-adrenergic blockade might have caused hypotension in the same mechanism.

On the other hand, vasopressin is a type of vasoactive agent that increases peripheral vasoconstriction via V1 receptors [3] and is commonly used to maintain vasoconstriction, particularly in distributive shock [1]. In our patient, severe hypotension immediately improved after administering vasopressin. This could be attributed to the fact that the mechanism action of vasopressin is different from that of catecholamines. Thus, vasopressin may be useful to support circulation in patients who have used α-adrenergic antagonists.

This is the first clinical case to describe unexpected hypotension as “catecholamine reversal.” Most antipsychotic agents have α-adrenergic blockade; thus, this educational case highlights that we should determine which vasoactive agent should be selected for the patient who uses these medicines.

## Conclusions

We described the first clinical case of “catecholamine reversal” and highlighted that if unexpected hypotension occurs in response to catecholamine infusion, we should suspect that the patient has used α-adrenergic antagonists. In such a situation, we should consider administration of vasopressin instead.
